# Work Addiction, Obsessive-Compulsive Personality Disorder, Burn-Out, and Global Burden of Disease: Implications from the ICD-11

**DOI:** 10.3390/ijerph17020660

**Published:** 2020-01-20

**Authors:** Paweł A. Atroszko, Zsolt Demetrovics, Mark D. Griffiths

**Affiliations:** 1 Department of Psychometrics and Statistics, Institute of Psychology, University of Gdańsk, Bażyńskiego 4, 80-952 Gdańsk, Poland; 2 Department of Clinical Psychology and Addiction, Institute of Psychology, ELTE Eötvös Loránd University, Izabella utca 46., H-1064 Budapest, Hungary; demetrovics.zsolt@ppk.elte.hu; 3 Psychology Department, Nottingham Trent University, 50 Shakespeare Street, Nottingham NG1 4FQ, UK; mark.griffiths@ntu.ac.uk

**Keywords:** burn-out, global burden of disease, mental health, obsessive-compulsive personality disorder, perfectionism, World Health Organization, work addiction, workaholism

## Abstract

Occupational stress and high workload are being increasingly recognized as significant contributors to the diseases and disorders constituting major components of the global burden of disease. A more detailed definition of burn-out was recently included by the World Health Organization (WHO) in the eleventh revision of the *International Classification of Diseases* (ICD-11) which reflects a growing acknowledgment of the role of professional work in mental health. One of the symptoms of obsessive-compulsive personality disorder/anankastic personality disorder (OCPD/APD) is an undue preoccupation with productivity to the exclusion of pleasure and interpersonal relationships. This compulsive overworking is closely related to the concept of work addiction, and OCPD/APD was suggested to be its major risk factor. OCPD/APD is the most prevalent personality disorder and one that appears to produce the highest direct and indirect medical costs. At the same time, it is vastly understudied. In recent years, it has been repeatedly emphasized that it requires consistent conceptualization and clarification of its overlapping with similar conditions. Even though the limited existing studies suggest its strong relationship with burn-out and depression among employed individuals, there has been no systematic effort to investigate its role in the consequences of occupational stress and high workload. This paper identifies several substantial gaps in the current understanding of the relationships between work addiction, OCPD/APD, burn-out, and the global burden of disease within the context of the WHO’s plan of developing evidence-based guidelines on mental wellbeing in the workplace.

## 1. Introduction

Occupational stress and high workload are being increasingly recognized as significant contributors to the diseases and disorders constituting major components of the global burden of disease. In this viewpoint paper, we analyze the substantial gaps in the current understanding of the relationships between work addiction, obsessive-compulsive personality disorder/anankastic personality disorder (OCPD/APD), burn-out and global burden of disease within the context of the World Health Organization’s (WHO) plan of developing evidence-based guidelines on mental wellbeing in the workplace. While the WHO focuses on meso-level interventions in the workplace in order to reduce risks of burn-out, the present paper suggests that more attention needs to be devoted to micro-level vulnerabilities such as rigid perfectionism underlying OCPD/APD and being closely related to work addiction, and their interactions with macro-level state policies, as well as meso-level organizational factors. As such, this paper is an attempt at delineating major areas of focus in compulsive overworking research and their interrelationships. It draws attention to the fact that work-related risk factors for burn-out and health identified by WHO mostly do not account for self-employment and individual vulnerabilities, and it suggests that meso-level organizational factors mediate between macro-level policies (and cultural values) and the micro-level individual vulnerabilities and compulsive overworking. Compulsive personality traits conducive to an undue preoccupation with productivity to the exclusion of pleasure and interpersonal relationships have been included in the official classifications of disorders since mid-20th century, and OCPD/APD is officially recognized since the second edition of the *Diagnostic and Statistical Manual of Mental Disorders* (DSM-2) and eighth revision of the *International Classification of Diseases* (ICD-8).

In this paper, we identify OCPD/APD as a formally and broadly recognized clinical entity and as a point of departure and reference for the analysis of the nature of compulsive overworking, and relate it to the current knowledge concerning work addiction. While organizational literature relatively widely recognizes so-called ‘workaholism’ as a dysfunctional and pathological form of heavy work investment constituting a significant problem for the functioning of employees and organizations, most research in the organizational framework refrains from explicit clinical frameworks in describing this phenomenon. Recent developments in the behavioral addiction field, and particularly within work addiction research, has allowed for greater integrations and consensus agreements regarding this addiction as a potential candidate to be officially recognized in classifications of disorders. Within the context of the increasing recognition of the role of mental health in the workplace, we suggest that it is timely to integrate organizational and clinical theoretical models and research results on compulsive overworking in order to develop the optimal strategies to prevent and treat this problematic behavior.

**Figure 1:**
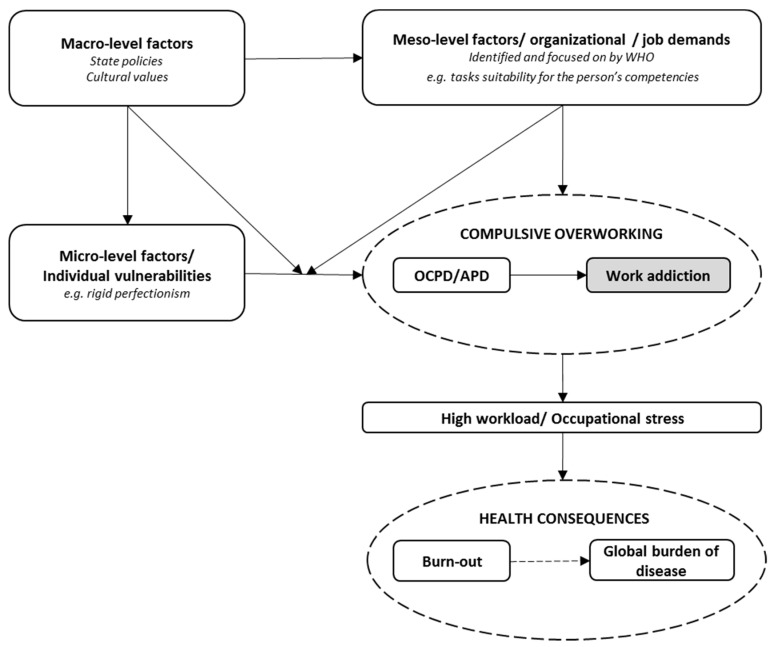
A model of the relationships between micro-, meso-, and macro- level factors, compulsive overworking (obsessive-compulsive personality disorder/anankastic personality disorder (OCPD/APD) and work addiction), high workload and occupational stress, and health consequences (burn-out and the global burden of disease). Dashed ellipses represent issues that require conceptual clarification.

Figure 1 depicts a simplified model of the described relationships and may be useful in identifying significant gaps in our knowledge on compulsive overworking. For clarity reasons, it does not explicitly represent all potential direct effects, such as (for example) the direct effect of compulsive overworking or meso-level factors on health consequences. Neither does it represent all potential effects such as the impact of micro-level factors or compulsive overworking on meso-level factors, which is a whole separate area of research that cannot be covered in much detail here. We believe that further integrations and developments require systematic efforts and wider networks of collaborations, especially between experts in work and organizational psychology, management and economics, and clinical psychologists, psychiatrists, and other health professionals, particularly specializing in occupational health and addictions.

A more detailed definition of burn-out was recently included by the WHO [1] in the eleventh revision of the *International Classification of Diseases* (ICD-11). The more comprehensive description from that in the ICD-10 reflects progress in research comprising a growing empirical evidence-base on the problem, and the fact that in recent years it has been viewed as an “epidemic” in specific professions including medical doctors and other health professionals [2,3,4]. Burn-out is defined as an occupational phenomenon, *“a syndrome conceptualized as resulting from chronic workplace stress that has not been successfully managed”* (p. 1) but is not classified as a medical condition. The announcement on the WHO webpage was followed by the statement that the *“World Health Organization is about to embark on the development of evidence-based guidelines on mental well-being in the workplace”* (p. 1).

A recently posted information sheet entitled ‘Mental health in the workplace’ contains further details on recognized work-related risk factors for health, and creating a healthy workplace which is described as *“one where workers and managers actively contribute to the working environment by promoting and protecting the health, safety and well-being of all employees”* (p. 1). The WHO’s ‘Global Plan of Action on Worker’s Health’ (2008–2017) and ‘Mental Health Action Plan’ (2013–2030) outline relevant principles, objectives, and implementation strategies to promote good mental health in the workplace [5]. The WHO recognizes that a negative working environment may lead to physical and mental health problems, harmful use of substances or alcohol, absenteeism, and lost productivity which, when taken altogether, create large detrimental psychosocial and economic costs. This is a noteworthy and highly welcomed effort which reflects a growing awareness of the multiplicity of factors that make professional work one of the main contributors to the global burden of disease [6,7,8,9,10]. However, the work-related risk factors for health that have been identified (meso-level factors in Figure 1), and the definition of healthy workplace do not recognize in any specific form the problematic behavior related to compulsive overworking and its idiosyncrasies which may have profound effects on health at the population level. There appears to be several reasons that it should, and that further research into obsessive-compulsive personality disorder/anankastic personality disorder (OCPD/APD) and work addiction could benefit from systematic support by the WHO.

## 2. OCPD/APD: The Most Prevalent and Understudied Personality Disorder

Obsessive-compulsive personality disorder (OCPD; DSM classification [11,12,13,14]) or anankastic personality disorder (APD; ICD classification) is the most prevalent personality disorder among the general population (3%–8%) and outpatient groups. It co-occurs with anxiety disorders, affective disorders, and substance-related disorders. OCPD/APD has been identified as producing the highest economic burden among personality disorders in terms of direct medical costs and productivity losses, even exceeding the costs of borderline personality disorder (BPD) [15]. Furthermore, patients with personality disorders have more extensive histories of psychiatric outpatient, inpatient, and psychopharmacologic treatment than do comparison patients with a major depressive disorder [16]. A large majority of OCPD/APD patients show rigid perfectionism. Currently, the WHO recognizes APD as personality disorder with prominent anankastic features (anakastia) in the ICD, with symptoms reflecting an excessive conscientiousness, scrupulousness, and undue preoccupation with productivity to the exclusion of pleasure and interpersonal relationships. While OCPD/APD is clearly associated with compulsive overworking, and some isolated studies indicate that it is a strong predictor of burn-out, especially the exhaustion component [17], there has been no systematic research into its role in occupational stress and burn-out.

Moreover, this disorder is still poorly understood, and the available data are often confusing and contradictory. The nomenclature itself is baffling, and demonstrates that the DSM model in literature on this issue is overwhelmingly predominant in the research because a *Web of Science* search on 8 June 2019, by the first author for papers published between 1900 and 2019 yielded eight results on ‘anankastic personality disorder’ and 339 on ‘obsessive-compulsive personality disorder’. In comparison, attention-deficit hyperactivity disorder yielded more than 32,000 results, which demonstrates how much attention is devoted to OCPD/APD. BPD, which is evaluated to produce similar economic costs to OCPD/APD, yielded more than 10,200 results, which constitutes a 30-fold difference in high-quality scientific publication coverage.

Recent review papers have emphasized that OCPD/APD needs consistent conceptualization and studies undertaken that separate its genuine co-occurrence with other disorders from the overlapping due to unclear distinction of OCPD/APD from similar conditions [13,14]. At present, there is very little that can be conclusively said about OCPD/APD, let alone its relationship with work addiction and burn-out. While work addiction is currently not formally recognized as a disorder in official psychiatric classifications, its considerable similarities to the OCPD/APD point to a need for further studies to clarify both concepts and their relationship. Moreover, a *Web of Science* search yields almost 50% more papers for ‘work addiction’ or ‘workaholism’ search in comparison to OCPD/APD, most of them appearing in the past 10 years [18]. Currently, there is a greater interest and more rapidly developing investigation into addictive properties of compulsive overworking than to its aspects related to disordered personality.

## 3. Current Status of Work Addiction Research and Its Relationship with OCPD/APD

The substantial increase in peer-reviewed papers concerning work addiction resulted in a recent unprecedented debate on the current status of this problematic behavior. It gave rise to consensus agreements among leading experts researching in the field regarding work addiction [18,19,20,21,22,23,24,25,26,27]. Most importantly, all of the experts viewed compulsive overworking as a genuine problem. Secondly, all the experts agreed that there was enough empirical data to support its relationship with impaired psychosocial functioning of clinical relevance. Furthermore, it was agreed that compulsive over-working was not a transient behavioral pattern and that there was evidence for the persistence of work addiction among a minority of individuals. Currently, most consistent available estimates suggest a prevalence of up to 10% being at risk of work addiction across industrialized countries (depending upon the instrument used) which is nontrivial and significantly higher than most of the other addictions. Finally, there was general agreement that the factors that contribute to work addiction go far beyond personality alone and that more research on the contribution of meso-level and macro-level factors to work addiction is urgently needed.

What is not known currently is how many work addicts there are across countries and professions, what are the most valid diagnostic symptoms, and how similar to other addictions it is. Furthermore, there is a lack of good quality data on the strength of the relationship between work addiction and health, particularly the extent to which work addiction is an independent risk factor for specific diseases and disorders, and which of the individual, organizational, social, and cultural factors most strongly contribute to this addiction. Based on the existing empirical data and conceptualizations, a general definition of work addiction as a behavioral addiction has been proposed, alongside suggestions concerning the development of specific diagnostic symptoms [27]. It is envisaged that this will facilitate studies on both the valid diagnostic criteria and more precise prevalence estimates of those at risk of work addiction.

One of the symptoms of OCPD/APD is an undue preoccupation with productivity to the exclusion of pleasure and interpersonal relationships which is strictly related to the way work addiction is conceptualized. Regarding the differences, there are a few major observations and arguments made recently, which point to the urgent need for further studies on this issue [27,28,29,30]. It has been noted that work addicts appear to present clear addiction symptoms such as loss of control over the behavior, and withdrawal, neither of which are defining criteria for OCPD/APD. Moreover, not all cases of work addiction are related to rigid perfectionism which underlies most occurrences of OCPD/APD. In recent studies on fairly large samples of working individuals in different countries, work addiction has been consistently related to attention-deficit hyperactivity disorder (ADHD) and more so than to obsessive-compulsive disorder (OCD) [31,32]. This parallels some previous clinical observations based on hundreds of case reports which have distinguished different types of work addicts [29,33]. These findings suggest that work addiction, similar to other addictions, including substance-related ones, may be a result of initial behavioral coping mechanism to deal with some other underlying psychopathology, and it may be specific for particular disorders such as OCPD/APD or ADHD. If that is the case, perhaps a re-evaluation of OCPD/APD and a more detailed approach to its diagnosis is needed.

## 4. OCPD/APD, Work Addiction, Burn-Out, and the Global Burden of Disease

Depression is among the most common causes of working disability in industrialized countries. A Finnish study showed that 50% of men and 28% of women with first-episode depression among employed individuals recruited from occupational health care units were diagnosed with OCPD/APD [34]. This is consistent with the effect sizes reported for the relationship between OCPD/APD and burn-out [17]. The current estimated cost of depression related to stress at work in the European Union is €617 billion annually [35], which is more than the gross domestic product (GDP) of most European countries [36]. Neuropsychiatric disorders and non-communicable diseases such as cardiovascular disease (CVD) and diabetes are among the leading causes of the global burden of disease [37]. Their total costs related to work stress are more than alarming, with the second most-costly category being CVD [35].

When taking into account the available data concerning the potential physiological mechanisms associating burn-out with CVD [38], well-evidenced association between depression and CVD [39] and especially with prognosis after acute coronary syndrome [40], overlapping of burn-out and depression [41], and the relationship between OCPD/APD and depression [11], it is surprising that there is so little research examining OCPD/APD, burn-out, and depression as independent risk factors for CVD. Moreover, it was recently argued that a high workload and its determinants, including potential genetic vulnerabilities, could be a substantial unaccounted confounding factor in studies related to the association between CVD and stimulant consumption, such as caffeine, the most widely used legal stimulant worldwide [42].

Work addiction has been associated with (mostly in cross-sectional studies) chronic stress in and outside work, burn-out and depression, as well as cardiovascular disease (CVD), anxiety, and other health problems (see Griffiths et al. [19]). However, very few largescale [31], high-quality epidemiological studies so far have evaluated it as an individual risk factor for specific diseases and disorders which contribute the most to the global burden of disease [37]. The association of OCPD/APD and work addiction with burn-out appears particularly important in this context because burn-out is a likely end-state of prolonged OCPD/APD and work addiction. Moreover, burn-out is a potential mediator between OCPD/APD/work addiction, and other health problems such as CVD or substance abuse—with which burn-out, as well as long working hours, in general—have been previously associated [43,44,45,46,47] (the path between burn-out and other disorders is represented by a dashed line in Figure 1 because currently we do not have enough data to conclude whether burn-out is a separate clinical entity, a risk factor for [40], or a component of other diseases and disorders [41]).

In this context, it appears that psychiatric work-related risk factors of non-communicable diseases, particularly OCPD/APD and work addiction, are a substantial, mostly undeveloped area of research that awaits systematic studies and integration with the existing frameworks. This also constitutes a special case of neglecting the role of mental illness in other health conditions [48]. There has been a significant effort to raise awareness about the association between mental and physical health in recent years, especially within the context of the global burden of disease, and the present paper constitutes yet another step in increasing recognition of the role of psychiatric disorders in general wellbeing of nations.

## 5. Work-Related Risk Factors for Health Mostly Do Not Account for Self-Employment and Individual Vulnerabilities

In the recent information sheet, the WHO [5] enumerated recognized work-related risk factors for health (meso-level factors in Figure 1): (i) Inadequate health and safety policies, (ii) poor communication and management practices, (iii) limited participation in decision-making or low control over one’s area of work, (iv) low levels of support for employees, (v) inflexible working hours, (vi) unclear tasks or organizational objectives, (vii) unsuitable tasks for the person’s competencies or a high and unrelenting workload, (viii) personal risks, (ix) lack of team cohesion or social support, and (x) bullying and psychological harassment (also known as “mobbing”). Many of these risk factors are well-established causes of professional burn-out, and most are associated with health via the mediating role of high and chronic stress.

Long working hours and stress (including occupational stress) are among the most well-recognized risk factors for a host of diseases and disorders including CVD [6], cancer [7], depression and anxiety [8], diabetes [9], and substance use and abuse [10]. However, neither epidemiological studies nor the WHO include or specifically recognize the self-imposed ‘unrelenting workload’ with which compulsive workers burden themselves. This is partially the reason why there are no adequate estimates of the extent to which long working hours are determined by specific life circumstances and basic needs, and to what extent they are driven by a compulsion to work.

The recognized risk factors, interventions, and good practices that protect and promote mental health in the workplace suggested by the WHO [5] could theoretically be exhaustive, meaning that a perfect implementation would completely reduce risks. However, this would only be true if two assumptions are made: (i) That every working professional is a member of an organization, and that (ii) there are only risk factors related to the work structure and work environment which have to be managed. However, it can be argued that these are unrealistic assumptions. For example, according to Eurostat [49], there were more than 32 million self-employed individuals in the European Union accounting for 14% of total employment, with almost one in every three individuals in Greece being self-employed in 2018 (30%), and around one in five in Italy (22%), Poland (18%), and Romania (17%). This means that such individuals manage their own work behaviors. Therefore, any policy solution would have to include their specific circumstances. Secondly, individual psychological factors such as personality and values are well-recognized determinants of work behaviors [25], and they have been associated with OCPD/APD [50] and work addiction [19]. The most notable association appears to be with rigid perfectionism, which is gaining more attention as a transdiagnostic process in the literature concerning psychopathology [51]. While research on transdiagnostic approaches is still preliminary and does not yet allow for a paradigm shift in classification and clinical care [52,53], it is a promising alternative to the current categorical diagnose-based psychiatric classification.

From this perspective, the systematic investigation of the relationship between OCPD/APD and work addiction may prove fertile ground for understanding the symptomatology of compulsion. There are also recognized genetic factors related to personality, including conscientiousness, which has been associated with OCPD/APD and work addiction [54], suggesting that some problematic features of work-related behaviors may be unaccounted by environmental interventions which do not recognize their nature. Without specific efforts and policies which identify behavioral patterns related to compulsive overworking, and aim at reducing it, even an individual provided with proper management, participation in decision-making, and high control over work (high control over work activities is, in fact, typical for OCPD/APD and work addiction) [27], support and flexible working hours, clear and suitable tasks, cohesive team, no personal risks, and no bullying or mobbing, could still compulsively work longer hours than required driven by dysfunctional perfectionism.

The need for perfection is not a problem in itself. However, its obsessive realization can have profound consequences for the health of the individual and their family members, especially children, as well as to the recipients of their work. Meta-analysis has shown a medium-to-large positive relationship between perfectionistic concerns and burn-out [55]. Among medical doctors, burn-out increases the risk of medical errors [56]. In such specific cases, the need for perfection of an individual becomes a public health concern. This is even more applicable to self-employed compulsive overworking individuals who theoretically should be the ones responsible for implementing their own good practices in their work. One study showed that across European countries, self-employed individuals experience more work-family conflict (WFC) than employees [57]. Moreover, among self-employed individuals, WFC cannot be explained by the level of state support (i.e., leave and childcare).

Countries with relatively substantial state support such as Sweden also score high on experienced WFC. This result points to unaccounted factors which may include compulsive overworking and which require further studies, especially given that work addiction was found to be significantly higher among self-employed individuals in a large nationally representative sample [58]. It appears to be a very unrealistic assumption that self-employed workers will self-manage their addiction, especially if it is taken into account that more than 90% of addicts never recognize their problem or seek help [59]. Furthermore, there is the issue of how to implement good practices, especially if most work addicts are unaware of their problematic behaviors. Work addiction is a highly specific problem which requires a highly specific approach. In this context, the WHO’s objective to support people with mental disorders at work needs to account for the specificity of work addiction as a potential mental health problem.

## 6. The Mediating Role of Organization Level Factors

The impact of organizational climate and values on employee work addiction understood as a negative phenomenon has been described and investigated since the late 1980s, most notably with Schaef and Fassel’s [60] idea of “addictive organizations” [61,62]. Currently, an increasing number of studies confirm that these factors may affect employee work addiction [63,64,65], including the mediating effect of work addiction between work stressors and health [66] (please note that meso-level factors in Figure 1 can also be conceptualized in terms of job demands and occupational stress). There is a consistent association between high job demands and work addiction [67] and overworking [68], including longitudinal data suggesting that high demands increase work addiction [69]. This is congruent with a model in which work addiction, similar to other addictions, is a result of ineffective coping with other underlying problems and stress [29,30,70], and at the same time generating more stress and problems. For example, high workload and burnout have been found to be associated with substance abuse [10,43,44].

Exacerbation of compulsive overworking could be a first response to high demands at work, followed by burn-out, and substance abuse, and leading to serious health problems. The role of job demands in relation to work addiction requires more longitudinal (and perhaps experimental) studies as some cross-sectional models and studies suggest that high job demands may be a consequence of work addiction [71]. Possible feedback loops (e.g., work addiction may increase work-role conflict and this, in turn, may increase work addiction) can explain such results and are theoretically feasible. Consequently, this issue requires more in-depth analysis. At the same time, it is necessary to remember that the environmental factors may have a more limited role in the cases of individuals highly predisposed to addiction [72], such as those showing the symptoms of OCPD, ADHD, or Type A personality [29,30,73] (therefore, the suggested moderating effects of macro-level and meso-level factors on the relationship between micro-level vulnerabilities and compulsive overworking). For this reason, individual vulnerabilities (such as rigid perfectionism) are likely to be the most important risk factor for this addiction, and the one responsible for the persistence of the disorder in the case of a minority of individuals.

Strategies to promote good mental health in the workplace need to account for individual risk factors for compulsive overworking, especially among self-employed individuals. Moreover, these micro-level factors interact with macro-level factors such as governmental pressures on productivity, economic growth, and innovation, as well as cultural factors related to consumerism, and personal and social focus values. The WHO’s recommendations based on recognized risk factors concentrate on organizational level (meso-level) interventions. However, it can be argued that this is to a large extent a mediating level between macro-level demands and micro-level vulnerabilities and compulsive overworking, and without changes on the macro-level (e.g., governmental policies) interventions on the lower levels will be to a significant extent limited in effectiveness. A special case is where there is no organizational level in-between. For example, if the government allows medical doctors to work an unlimited number of hours under self-employment, it can be expected that individuals with particular vulnerabilities may devote an inordinate amount of effort to work despite evident serious negative consequences for their and/or their families psychosocial functioning (moderating effect of macro-level policies in Figure 1). In fact, this has been recently recognized by the Doctors’ Trade Union of Poland as one of the factors involved in physicians’ death cases due to long working hours [74].

It has been demonstrated that the prevalence of a disease or disorder in a population has a relationship with the average level of behaviors related to that disease/disorder within the population [75]. This epidemiological observation indicates that the number of compulsively overworking individuals reflects population work-related behaviors. In recent decades across Asia, numerous countries have experienced rapid economic growth, and at the same time, there has been an increase in overwork-related deaths and suicides, together with other indicators of the decline in health and wellbeing due to work overload [76]. This does not indicate that economic growth causes poorer health, but suggests that imbalanced, fast, and resilient development of an economy without regard for human wellbeing may result in severe health consequences and associated nontrivial economic costs. Pressures on economic growth are closely related to state policies. Consequently, interventions aimed at improving the health of the workforce need to be directed at governments, and this lays in the competencies of organizations such as the WHO. Such interventions should not infringe personal freedoms. However, raising awareness about the enormous costs on personal, social, and economic level related to excessive work could be beneficial to both individuals and whole state economies through the reduction of medical costs and improvement in productivity.

## 7. Conclusions

It is postulated that the existing empirical data provides enough substantiation to support systematic efforts from the WHO in promoting and enabling further investigations on the relationships between work addiction, OCPD/APD, burn-out, and the global burden of disease. This would facilitate progress in the field if work addiction and OCPD/APD are included among the specific action points on the WHO’s agenda in their implementation of the ‘Mental Health Action Plan’ (2013–2030). A further advanced investigation in this area requires more resources, including funding schemes and other incentives. However, most importantly, it would benefit from more interest and involvement of a wide range of specialists from different fields and areas of research, most importantly from psychiatry, but also from epidemiology, public health, education, economics, and sociology. Particularly, clinical and organizational frameworks in the research on compulsive overworking require systematic integration and developments.

New, interesting, and significant studies on previously neglected aspects of work addiction appear, such as aggressive behavior of work addicts in the workplace [77] which can be conceptualized as an addiction symptom related to harm to oneself and/or others [27], and easily integrated with clinical frameworks. However, one of the main challenges identified within the recent debate on the current status of work addiction research is the low quality of a majority of studies on this problematic behavior. This parallels (to some extent) conclusions from the review papers on OCPD/APD, which mainly point to the conceptual vagueness of the construct. As a formally recognized entity both in DSM and ICD classification systems, and the most prevalent personality disorder, it also appears to be a much neglected problem. More studies are needed on: (i) Work addiction and OCPD/APD as individual risk factors for burn-out and depression, as well as other diseases and disorders which constitute major components of global burden of disease, notably CVD, cancer, diabetes, anxiety disorders, and substance use disorders, (ii) how to validly identify compulsive over-workers in the workplace and outside, and to what extent work addiction is similar to other addictions, and (iii) which factors influence compulsive overworking (especially in the workplace context) and how to develop good practices and interventions which can potentially reduce it.

Systematic support from the WHO would draw attention to this area of research and promote improvement in the quality of studies, as well as facilitate a higher integration of the results. This is consistent with the objectives of the WHO’s ‘Mental Health Action Plan’ (2013–2030) which includes strengthening information systems, evidence, and research for mental health, with such cross-cutting principles as evidence-based practice and a multi-sectoral approach. The absence of evidence is not evidence of absence. Work addiction and OCPD/APD are genuine but much under-investigated problems that result in their current neglect by health institutions and policymakers.
